# Effectiveness of montelukast administered as monotherapy or in combination with inhaled corticosteroid in pediatric patients with uncontrolled asthma: a prospective cohort study

**DOI:** 10.1186/1710-1492-10-21

**Published:** 2014-05-06

**Authors:** Denis Bérubé, Michel Djandji, John S Sampalis, Allan Becker

**Affiliations:** 1 CHU Ste-Justine, Université de Montréal, Montréal, Québec, Canada; 2 Merck Canada Inc., Kirkland, Québec, Canada; 3 Current address: Novartis Canada, Dorval, Québec, Canada; 4 McGill University, Montréal, Québec, Canada; 5 JSS Medical Research, Montréal, Québec, Canada; 6 Section of Allergy and Clinical Immunology, Department of Pediatrics and Child Health, University of Manitoba, Manitoba, Canada

**Keywords:** Asthma, Montelukast, Inhaled corticosteroids, Pediatric, Preschool age, School age

## Abstract

**Background:**

Asthma is the most common chronic disease of childhood and a leading cause of childhood morbidity. The aim of the current study was to assess the effectiveness of montelukast administered as monotherapy or in combination with current inhaled corticosteroids (ICS) in pediatric patients with uncontrolled asthma as per the Canadian Asthma Consensus Guidelines.

**Methods:**

Twelve-week, multicentre, open-label, observational study. Primary effectiveness outcome was the proportion of patients achieving asthma control (Asthma Control Questionnaire (ACQ) score ≤0.75) at weeks 4 and 12.

**Results:**

A total of 328 patients with uncontrolled asthma (ACQ > 0.75) were enrolled with mean ± SD age of 6.9 ± 3.4 years. Among these, 76 (23.2%) were treated with montelukast monotherapy and 252 (76.8%) with montelukast combined with ICS. By 4 weeks of treatment 61.3% and 52.9% of the patients in the monotherapy and combination group, respectively, achieved asthma control. These proportions increased to 75.0% and 70.9%, respectively, at 12 weeks. Within the monotherapy group, clinically significant improvements in the ACQ score (mean ± SD of 1.67 ± 0.69, 0.71 ± 0.70 and 0.50 ± 0.52 at baseline, 4 and 12 weeks, respectively; p < 0.001) and the PACQLQ score (mean ± SD of 5.34 ± 1.14, 6.32 ± 0.89 and 6.51 ± 0.85 at baseline, 4 and 12 weeks, respectively; p < 0.001) were observed. In the combination group, the mean ± SD ACQ score significantly improved from 2.02 ± 0.83 at baseline to 0.90 ± 0.86 at 4 weeks and 0.64 ± 0.86 at 12 weeks (p < 0.001), while the PACQLQ score improved from 4.42 ± 1.35 at baseline to 5.76 ± 1.30 at 4 weeks and 6.21 ± 1.03 at 12 weeks (p < 0.001). After a 12-week montelukast add-on therapy, 22.6% of patients reduced their ICS dosage. Similar results were observed among preschool- and school-aged patients.

**Conclusions:**

Montelukast as monotherapy or in combination with ICS represents an effective treatment strategy for achieving asthma control in pediatric patients and improving caregivers’ quality of life.

**Trial registration:**

This study is registered at ClinicalTrial.gov: NCT00832455.

## Background

Asthma is a chronic inflammatory disorder of the airways with a heterogeneous target age group and an initial diagnosis age of as early as infancy. Its prevalence, especially among children, is increasing worldwide [1,2], including Canada [3,4]. From 2000 to 2001, 13.4% of Canadian children aged up to 11 years old were diagnosed with asthma [3]. Relative to the 1994 to 1995 period, this represents a statistically significant increase in the asthma prevalence of nearly 70,000 diagnoses of asthmatic children [3], rendering asthma one of the most prevalent chronic conditions affecting Canadian children.

Current asthma treatment guidelines recognize the importance of early and aggressive intervention for asthma and recommend low-dose inhaled corticosteroids (ICSs) as first-line treatment in childhood [2,5-8]. However, despite ICS treatment, an important proportion of patients remain with uncontrolled asthmatic symptoms. In addition, the response to asthma therapy appears to be variable since some asthmatic children who do not respond to ICSs may respond to other therapies [9,10]. This further highlights the need to identify alternative treatment strategies that will expand the array of therapeutic options available to physicians who treat pediatric asthma [11].

Leukotriene receptor antagonists (LTRAs), such as montelukast, provide an alternative treatment for asthma patients who are not controlled or satisfied with ICS therapy [2,5-7,12]. Montelukast is an orally administered, once-daily LTRA that can be prescribed as monotherapy or in combination with other asthma medications, including ICSs, for the treatment of asthma.

Although results from controlled randomized clinical trials have provided evidence of the montelukast efficacy in the treatment of asthmatic children [13,14], continuous evaluation of the effectiveness and safety of montelukast in a less controlled real-life setting is essential to help health care professionals bridge the gap between current knowledge and routine practice in the management of asthmatic children. There is currently little information available on montelukast effectiveness in every day practice for children, which could complement the findings of randomized clinical trials. Therefore, the principal aim of this study was to assess the effectiveness of montelukast administered either as monotherapy or in combination with current ICS treatment in pediatric patients with uncontrolled asthma, in a clinical setting emulating real-life.

## Methods

### Study design

This was a 12-week, open-label, multicenter, prospective study conducted in 58 Canadian clinics between June 2006 and October 2008. Patients were treated with montelukast sodium for 12 weeks, either as a monotherapy or in combination with their current ICS treatment. Clinical assessments were conducted at baseline, 4 and 12 weeks of treatment at the clinics of their treating physicians. During the course of the study, tapering of ICS dosage was performed at the discretion of the treating physician and on an individual basis when asthma control was achieved. An optional visit after 8 weeks of treatment was performed to determine if an ICS dosage adjustment was necessary and to assess asthma control of patients previously tapered. Parents or legal guardians provided written informed consent prior to the participation of their children in this study. The study was approved by three independent Ethics Review Boards (IRB Services, Aurora, Ontario; the College of Physicians and Surgeons of Alberta, Edmonton; and the *Comité central de l’éthique de la recherche du Ministère de la santé et des services sociaux du Québec*, Montréal, Québec), and was conducted in accordance with ICH Good Clinical Practice Guidelines, the World Medical Association Declaration of Helsinki and all applicable local regulations.

### Patients

Eligible patients were between 2 and 14 years of age and had been diagnosed with asthma for at least 6 months. In order to be included in the study, patients had to have a peak expiratory Flow (PEF) ≥ 80% of the predicted value (applicable only for patients older than 7 years old) and they had to be either currently untreated, using a short-acting β_2_–agonist (SABA) on an as-needed basis or using an ICS at any dosage. In addition, one of the following conditions had to be satisfied: i) the physician and/or patient was dissatisfied with the current controller therapy; ii) the patient was reluctant to take ICS therapy, or; iii) the patient was insufficiently controlled with the current therapy through the preceding 6 weeks. Finally, eligible patients had to have uncontrolled asthma as per the 2003 Canadian Asthma Consensus Guidelines [6].

Patients were excluded if their asthma symptoms were controlled and if they were treated with montelukast or any of the following treatments at the time of entry into the study: long-acting β_2_-agonist (LABA) alone or in a combination product, oral prednisone, regular use of theophylline and/or other asthma medications such as sodium cromoglycate or nedocromil. Patients using an antibiotic for respiratory tract infection at the time of entry into the study or treated with an antibiotic for respiratory tract infection (initiation of antibiotic treatment was permitted during the study) within 30 days were also excluded. A history of cystic fibrosis, immune deficiency requiring specific therapy or any other disease that could influence the evolution of asthma was also a reason for exclusion. Finally, patients with a history of hypersensitivity to any component of montelukast were excluded.

Considering that the primary outcome measure was the proportion of patients achieving asthma control based on the ACQ criteria (ACQ ≤ 0.75), a re-analysis of the data was conducted including only patients with ACQ > 0.75 at baseline the results of which are reported here.

### Treatment strategies

All patients were treated with montelukast sodium (SINGULAIR®, Merck & Co. Inc., USA) taken once-daily at bedtime as monotherapy or in addition to their current ICS therapy. Patients aged between 6 and 14 years were treated with 5 mg montelukast sodium chewable tablets, while patients between 2 and less than 6 years of age were treated with 4 mg montelukast chewable tablets. The 4 mg granule formulation was also available for the latter age group on demand. The use of a short-acting β_2_-agonist (SABA) as rescue medication was allowed during the study, but patients were asked to refrain from its utilization for 6 hours prior each study visit.

### Outcome measures

The primary effectiveness outcome measures was the proportion of patients achieving asthma control, defined as a score ≤ 0.75 [15] in the self-administered Asthma Control Questionnaire (ACQ) (completed by the patient or their caregiver) [16]. Secondary effectiveness outcome measures included: (i) the mean change in ACQ score between baseline and the 4- and 12-week assessments, considering a change of ≥ 0.5 in ACQ score as clinically important [16]; (ii) the change in quality of life of the caregivers between baseline and the 4- and 12-week assessments, as assessed using the Pediatric Asthma Caregivers Quality of Life Questionnaire (PACQLQ) [17], considering changes of ≥ 0.7 in PACQLQ as clinically important [17]; (iii) the patient (completed by the patient or their caregiver) and physician satisfaction with treatment as measured using the 5-point Likert scale ranging from 0 (very dissatisfied) to 4 (very satisfied), upon 4 and 12 weeks of treatment with montelukast; and (iv) the proportion of patients on montelukast combination therapy whose baseline ICS daily dosage was tapered to a lower ICS dose category after 4, 8 and 12 weeks of treatment. The ICS daily doses were categorized according to the 2006 report of the Global Initiative for Asthma (GINA) [18] as follows: (i) low dose, defined as ≤200 μg/day of fluticasone propionate or equivalent (≤200 μg/day of beclomethasone dipropionate and ≤200 μg/day of budesonide); (ii) moderate dose, defined as >200 to ≤500 μg/day of fluticasone propionate or equivalent (>200 to ≤400 μg/day of beclomethasone dipropionate and >200 to ≤400 μg/day of budesonide); and (iii) high dose, defined as >500 μg/day of fluticasone propionate or equivalent (>400 μg/day of beclomethasone dipropionate and >400 μg/day of budesonide).

Compliance with the study medication was assessed by tablet counts, as recorded in the study worksheets. Safety and tolerability were assessed with the incidence of treatment-emergent adverse events, which were coded and reported according to the MedDRA dictionary of terms, version 9.0 [19].

### Statistical methods

Descriptive statistics were produced for patient demographics and characteristics at baseline. Comparisons between baseline and follow-up visits were performed with the matched Chi-Square test for categorical scales and the paired Student’s t-test for continuous scales. Two-tailed tests were performed using a significance level (α) of 0.05. Subgroup analyses by treatment strategy and stratified analyses for preschool-aged children (less than 6 years old) and school-aged children (6 years of age or older) were performed. There were no imputations for missing data. All analyses were performed using the SPSS version 12.0 for Windows (SPSS Inc., Chicago, IL).

## Results

### Patient disposition

A total of 420 patients with uncontrolled asthma as per the 2003 Canadian Asthma Consensus Guidelines completed the baseline assessment of whom 92 (21.9%) had an ACQ score of ≤ 0.75 at baseline. Considering that the primary outcome measure was the proportion of patients achieving asthma control based on the ACQ criteria, only the 328 patients with ACQ > 0.75 were included in this analysis. Among these, 320 (97.6%) and 288 (87.8%) patients completed the 4- and 12-week assessment, respectively, while the optional 8-week assessment was performed on 197 (60.1%) patients. There were 40 (12.2%) patients who were discontinued from the study: 10 (3.0%) due to an adverse event, 8 (2.4%) withdrew consent, 9 (2.7%) were lost to follow-up, 4 (1.2%) due to protocol violation, 8 (2.4%) discontinued for other reasons, while the reason of discontinuation was missing for 1 (0.3%) patient.

### Patient demographics and baseline characteristics

The demographics and baseline characteristics of the study population are summarized in Table 1. The mean (SD) age was 6.92 (3.35) years, 192 (58.5%) patients were male and 209 (63.7%) were Caucasian. At baseline, 252 patients were on ICS therapy and were therefore included in the montelukast add-on group, the majority of whom were taking moderate doses of ICS (n = 143; 56.7%). The remaining 76 patients were not taking ICS at baseline and comprised the montelukast monotherapy treatment group. Overall, at baseline, 269 (82.0%) patients had night-time symptoms ≥ 1 night/week, 247 (75.3%) had daytime symptoms ≥ 4 days/week, and 122 (37.2%) reported absenteeism from school in the last week due to asthma. Notably, SABA utilization (≥4 doses in the last week) was reported twice as frequently by patients in the combination therapy compared to the monotherapy group (66.7% vs 34.2%).

**Table 1:** Demographics and baseline characteristics

**Characteristics**	**Montelukast monotherapy**			**Montelukast + ICS**			**Total**
	**Preschool age**	**School age**	**All**	**Preschool age**	**School age**	**All**	
**N**	**34**	**42**	**76**	**112**	**140**	**252**	**328**
Age (years), mean (SD)	4.01 (1.12)	9.19 (2.26)	6.87 (3.19)	3.86 (1.13)	9.54 (2.35)	7.02 (3.41)	6.92 (3.35)
Gender, n (%)							
Male	18 (52.9)	25 (59.5)	43 (56.6)	60 (53.6)	89 (63.6)	149 (59.1)	192 (58.5)
Female	16 (47.1)	17 (40.5)	33 (43.4)	52 (46.4)	51 (36.4)	103 (40.9)	136 (41.5)
Race, n (%)							
Caucasian	25 (73.5)	32 (76.2)	57 (75.0)	66 (58.9)	86 (61.4)	152 (60.3)	209 (63.7)
Black	0 (0.0)	1 (2.4)	1 (1.3)	8 (7.1)	13 (9.3)	21 (8.3)	22 (6.7)
Asian	4 (11.8)	8 (19.0)	12 (15.8)	32 (28.6)	32 (22.9)	64 (25.4)	76 (23.2)
Hispanic	0 (0.0)	1 (2.4)	1 (1.3)	3 (2.7)	3 (2.1)	6 (2.4)	7 (2.1)
Other	3 (8.8)	0 (0.0)	3 (3.9)	2 (1.8)	6 (4.3)	8 (3.2)	11 (3.4)
Missing	2 (5.9)	0 (0.0)	2 (2.6)	1 (0.9)	0 (0.0)	1 (0.4)	3 (0.9)
Duration of asthma since diagnosis (years), mean (SD)	2.06 (1.11)	4.32 (3.25)	3.31 (2.76)	2.11 (1.27)	5.46 (3.08)	3.97 (2.96)	3.82 (2.92)
Smoking history, n (%)							
Patient is a smoker	0 (0.0)	0 (0.0)	0 (0.0)	0 (0.0)	0 (0.0)	0 (0.0)	0 (0.0)
Patient quit smoking	0 (0.0)	0 (0.0)	0 (0.0)	0 (0.0)	1 (0.7)	1 (0.4)	1 (0.3)
Patient never smoked	34 (100.0)	41 (97.6)	75 (98.7)	104 (92.9)	133 (95.0)	237 (94.0)	312 (95.1)
Member of household is a smoker	8 (23.5)	14 (33.3)	22 (28.9)	20 (17.9)	40 (28.6)	60 (23.8)	82 (25.0)
Member of household quit smoking	5 (14.7)	11 (26.2)	16 (21.1)	8 (7.1)	7 (5.0)	15 (6.0)	31 (9.5)
Use of ICS at baseline, n (%)							
Low dose^*^	-	-	-	58 (51.8)	39 (27.9)	97 (38.5)	97 (29.6)
Moderate dose^†^	-	-	-	53 (47.3)	90 (64.3)	143 (56.7)	143 (43.6)
High dose^‡^	-	-	-	1 (0.9)	11 (7.9)	12 (4.8)	12 (3.7)
Profile of asthma symptoms, n (%)							
1. Daytime symptoms ≥ 4 days/week	24 (70.6)	26 (61.9)	50 (65.8)	91 (81.3)	106 (75.7)	197 (78.2)	247 (75.3)
2. Night-time symptoms ≥ 1 night/week	29 (85.3)	30 (71.4)	59 (77.6)	99 (88.4)	111 (79.3)	210 (83.3)	269 (82.0)
3. Absenteeism from school due to asthma in the last week	7 (20.6)	16 (38.1)	23 (30.3)	33 (29.5)	66 (47.1)	99 (39.3)	122 (37.2)
4. SABA ≥ 4 doses in the last week^§^	13 (38.2)	13 (31.0)	26 (34.2)	76 (67.9)	92 (65.7)	168 (66.7)	194 (59.1)
5. FEV in one second or PEF ≥90% of their personal best in the last week	2 (5.9)	9 (21.4)	11 (14.5)	11 (9.8)	41 (29.3)	52 (20.6)	63 (19.2)
6. Diurnal variability in peak expiratory flow >10% to 15% in the last week	2 (5.9)	2 (4.8)	4 (5.3)	8 (7.1)	22 (15.7)	30 (11.9)	34 (10.4)

*Low dose was defined as ≤ 200 μg/day for fluticasone propionate or equivalent (≤200 μg/day for beclomethasone dipropionate and ≤200 μg/day for budesonide).

^†^Moderate dose was defined as >200 to 500 μg/day for fluticasone propionate or equivalent (>200 to 400 μg/day for beclomethasone dipropionate and >200 to 400 μg/day for budesonide) [18].

^‡^High dose was defined as > 500 μg/day for fluticasone propionate or equivalent (>400 μg/day for beclomethasone dipropionate and >400 μg/day for budesonide).

^§^Excluding one dose/day before exercise.

### Effectiveness outcomes

Table 2 presents the proportions of patients who achieved asthma control after 4 and 12 weeks of treatment with montelukast, administered either as a monotherapy or in addition to ICS therapy, overall and stratified by treatment strategy and age group. The overall proportion of patients who achieved asthma control (ACQ score ≤ 0.75) was 54.9% (n = 175) at 4 weeks and 71.9% (n = 207) at 12 weeks. Among preschool patients, the proportion of patients with controlled asthma increased from 63.3% (n = 88) at 4 weeks to 77.3% (n = 99) at 12 weeks, while among school aged patients, these proportions were 48.3% (n = 87) and 67.5% (n = 108), respectively. This significant rate of asthma control was consistent across both treating strategies; montelukast alone and in combination with ICS.

**Table 2:** Proportion of patients with asthma control (ACQ score ≤ 0.75)

**Asthma control**	**4 weeks**		**12 weeks**	
	**n**	**%**	**n**	**%**
**Montelukast monotherapy**				
**All patients, n (%)**	(N = 75)		(N = 68)	
Well controlled	46	61.3	51	75.0
Not controlled	29	38.7	17	25.0
**Preschool aged patients, n (%)**	(N = 34)		(N =32 )	
Well controlled	19	55.9	24	75.0
Not controlled	15	44.1	8	25.0
**School aged patients, n (%)**	(N =41 )		(N = 36)	
Well controlled	27	65.9	27	75.0
Not controlled	14	34.1	9	25.0
**Montelukast + ICS**				
**All patients, n (%)**	(N = 244)		(N = 220)	
Well controlled	129	52.9	156	70.9
Not controlled	115	47.1	64	29.1
**Preschool aged patients, n (%)**	(N = 105)		(N = 96)	
Well controlled	69	65.7	75	78.1
Not controlled	36	34.3	21	21.9
**School aged patients, n (%)**	(N = 139)		(N = 124)	
Well controlled	60	43.2	81	65.3
Not controlled	79	56.8	43	34.7
**Total study sample (Montelukast monotherapy & Montelukast + ICS)**				
**All patients, n (%)**	(N = 319)		(N = 288)	
Well controlled	175	54.9	207	71.9
Not controlled	144	45.1	81	28.1
**Preschool aged patients, n (%)**	(N = 139)		(N = 128)	
Well controlled	88	63.3	99	77.3
Not controlled	51	36.7	29	22.7
**School aged patients, n (%)**	(N = 180)		(N = 160)	
Well controlled	87	48.3	108	67.5
Not controlled	93	51.7	52	32.5

**Figure 1:**
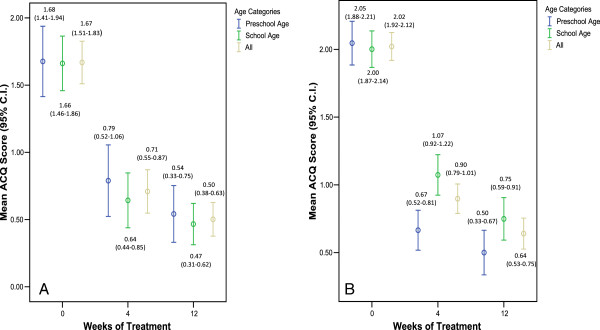
**Mean Asthma Control Questionnaire (ACQ) score over time. A)** Montelukast monotherapy; **B)** Montelukast + ICS.

The mean (SD) ACQ score of the total study sample decreased from 1.94 (0.82) at baseline to 0.85 (0.83) at 4 weeks and 0.61 (0.79) at 12 weeks of treatment, representing statistically and clinically significant absolute mean (SD) changes of −1.08 (1.00) and −1.34 (1.03) from baseline to 4 and 12 weeks, respectively (p < 0.001) (Table 3). Among the patients treated with montelukast monotherapy, the mean (SD) ACQ score significantly decreased from 1.67 (0.69) at baseline to 0.71 (0.70) at 4 weeks and to 0.50 (0.52) 12 weeks (Figure 1A). Among the patients treated with the montelukast add-on treatment strategy, the mean (SD) ACQ score significantly decreased from 2.02 (0.83) at baseline to 0.90 (0.86) at 4 weeks and to 0.64 (0.86) at 12 weeks (Figure 1B).

**Table 3:** Mean change in Asthma Control Questionnaire and Pediatric Asthma Caregivers Quality of Life Questionnaire

**Age group**		**Treatment group**				**Total**	
		**Montelukast monotherapy**		**Montelukast + ICS**			
		**Mean**	**SD**	**Mean**	**SD**	**Mean**	**SD**
**All patients**							
ACQ score*	Change between Week 4 and Baseline	−0.95	0.88	−1.12	1.03	−1.08	1.00
	Change between Week 12 and Baseline	−1.15	0.79	−1.40	1.09	−1.34	1.03
PACQLQ score*	Change between Week 4 and Baseline	0.98	1.12	1.34	1.34	1.25	1.30
	Change between Week 12 and Baseline	1.13	1.04	1.78	1.36	1.63	1.32
***Emotional function*** *	Change between Week 4 and Baseline	0.90	1.14	1.25	1.32	1.17	1.29
	Change between Week 12 and Baseline	1.02	1.01	1.71	1.41	1.55	1.36
***Activity limitation*** *	Change between Week 4 and Baseline	1.16	1.32	1.53	1.61	1.44	1.55
	Change between Week 12 and Baseline	1.38	1.34	1.94	1.51	1.81	1.49
**Pre-School patients**							
ACQ score*	Change between Week 4 and Baseline	−0.89	1.10	−1.38	1.08	−1.26	1.11
	Change between Week 12 and Baseline	−1.13	0.94	−1.56	1.16	−1.45	1.12
PACQLQ score*	Change between Week 4 and Baseline	1.01	1.19	1.73	1.42	1.55	1.40
	Change between Week 12 and Baseline	1.18	1.06	2.03	1.30	1.82	1.30
***Emotional function*** *	Change between Week 4 and Baseline	0.88	1.16	1.60	1.41	1.42	1.39
	Change between Week 12 and Baseline	1.05	0.95	1.94	1.34	1.72	1.31
***Activity limitation*** *	Change between Week 4 and Baseline	1.29	1.40	2.02	1.62	1.84	1.60
	Change between Week 12 and Baseline	1.47	1.47	2.24	1.45	2.05	1.49
**School aged patients**							
ACQ score*	Change between Week 4 and Baseline	−1.01	0.64	−0.93	0.94	−0.94	0.88
	Change between Week 12 and Baseline	−1.18	0.64	−1.27	1.02	−1.25	0.95
PACQLQ score*	Change between Week 4 and Baseline	0.96	1.08	1.04	1.20	1.02	1.17
	Change between Week 12 and Baseline	1.09	1.03	1.58	1.37	1.47	1.31
***Emotional function*** *	Change between Week 4 and Baseline	0.92	1.14	0.99	1.19	0.97	1.18
	Change between Week 12 and Baseline	1.00	1.08	1.53	1.44	1.41	1.38
***Activity limitation*** *	Change between Week 4 and Baseline	1.04	1.26	1.15	1.49	1.13	1.44
	Change between Week 12 and Baseline	1.29	1.23	1.70	1.52	1.61	1.47

*P < 0.001 for all changes from baseline based on Student’s t-test for Paired Observations.

The mean ACQ scores throughout the treatment period for the monotherapy and combination therapy patients, stratified by age group are also shown in Figure 1A and B. Among preschool and school-aged patients treated with montelukast monotherapy, the mean (SD) ACQ score significantly decreased from 1.68 (0.75) and 1.66 (0.65) at baseline to 0.79 (0.76) and 0.64 (0.65) at 4 weeks, to 0.54 (0.59) and 0.47 (0.45) at 12 weeks (Figure 1A), representing statistically and clinically significant absolute mean (SD) changes of −1.13 (0.94) and −1.18 (0.64) from baseline to 12 weeks, respectively (Table 3). Similarly, statistically and clinically significant decreases in the ACQ score were also observed among preschool and school aged patients treated with montelukast add-on therapy (Figure 1B and Table 3). Among children 7 years of age or older the PEF assessment showed a statistically significant improvement increasing from 253.9 L/min at baseline to 275.0 L/min at 12 weeks of treatment (P < 0.001).

**Figure 2:**
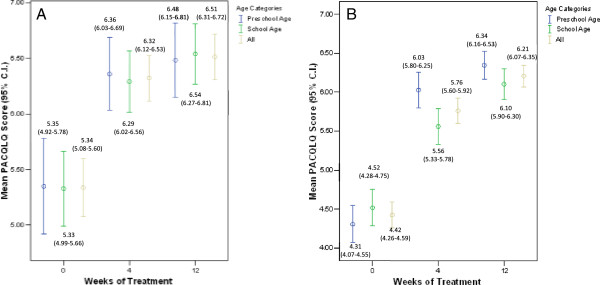
**Mean Pediatric Asthma Caregivers Quality of Life Questionnaire (PACQLQ) score over time. A)** Montelukast monotherapy; **B)** Montelukast + ICS.

**Figure 3:**
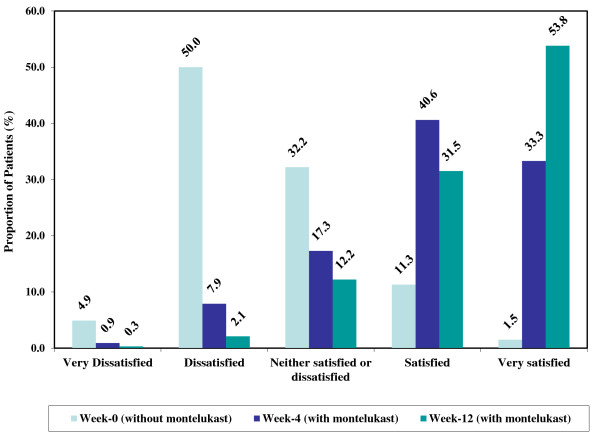
**Patient global satisfaction upon treatment with montelukast. ***Note: Percentages were calculated on available observations.*

**Figure 4:**
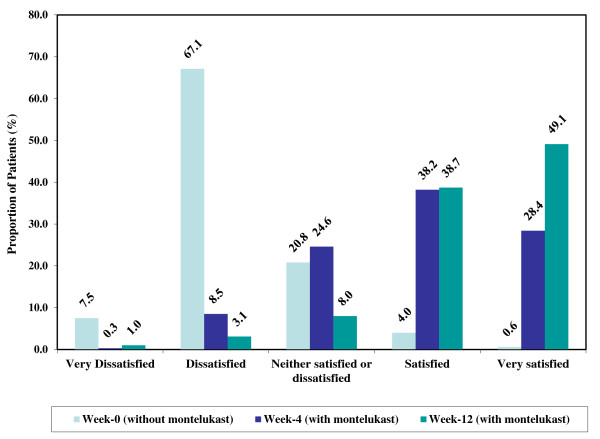
**Physician global satisfaction with utilization of montelukast. ***Note: Percentages were calculated on available observations.*

The mean (SD) PACQLQ score of the total study sample increased from 4.63 (1.36) at baseline to 5.89 (1.24) at 4 weeks (mean (SD) change = 1.55 (1.40); P < 0.001) and 6.28 (1.00) at 12 weeks (mean (SD) change = 1.82 (1.30); P < 0.001). Among the patients who adopted the montelukast monotherapy treatment strategy, the mean (SD) PACQLQ score increased from 5.34 (1.14) at baseline to 6.32 (0.89) at 4 weeks and 6.51 (0.85) at 12 weeks. The absolute mean (SD) change in PACQLQ of 0.98 (1.12) at 4 weeks and 1.13 (1.04) at 12 weeks was both clinically (change in PACQLQ > 0.7) and statistically significant (p < 0.001) (Table 3). Among the patients who adopted the montelukast add-on treatment strategy, the mean (SD) PACQLQ score increased from 4.42 (1.35) at baseline to 5.76 (1.30) at 4 weeks and 6.21 (1.03) at 12 weeks corresponding to a mean (SD) absolute change of 1.34 (1.34) at 4 weeks and 1.78 (1.36) at 12 weeks (p < 0.001) (Table 3). In both treatment strategies, significant changes were observed in both the emotional and activity limitation domains of the PACQLQ questionnaire. Comparable clinically and statistically significant changes in PACQLQ scores were observed among preschool and school aged patients (Figure 2A and B and Table 3).Figures 3 and 4 summarize the results of the patients and physicians global satisfaction with montelukast, respectively. At baseline, 54.9% of the patients were dissatisfied or very dissatisfied with their current asthma therapy and 12.8% were satisfied or very satisfied. After 4 and 12 weeks of treatment with montelukast, 8.8% and 2.4% of the patients were dissatisfied/very dissatisfied and 73.9% and 85.3% were satisfied/very satisfied, respectively. With regards to the physician’s global satisfaction, 74.6% of the treating physicians were dissatisfied or very dissatisfied with their patient’s current asthma therapy and 4.6% were satisfied or very satisfied at baseline. After 4 and 12 weeks of treatment with montelukast, 8.3% and 4.1% of the physicians were dissatisfied/very dissatisfied while 66.6% and 87.8% were satisfied/very satisfied, respectively. Overall, the changes in patient and physician satisfaction upon treatment with montelukast for 4 and 12 weeks were statistically significant (p < 0.001) without any significant differences between preschool and school aged patients (data not shown).

The proportions of patients who tapered their baseline ICS daily dosage use to a lower ICS dose category after adding montelukast to their treatment regimen are reported in Table 4. There were 45 (18.4%), 40 (25.2) and 44 (20.0) patients who reduced their ICS dosage after the addition of montelukast to their current ICS treatment regimen at 4, 8 and 12 weeks, respectively. Similar results were observed for preschool and school aged patients.

**Table 4:** Proportion of patients who tapered their dosage of inhaled corticosteroids in the montelukast add-on group

	**Duration of treatment**			
	**Baseline**	**4 weeks**	**8 weeks**	**12 weeks**
**All patients**	328	320	197	288
**Use of ICS, n**				
**Yes**	252	245	159	220
No	76	75	38	68
**Tapered ICS, n (%)**				
Yes^*^	-	45 (18.4)	40 (25.2)	44 (20.0)
No	-	194 (79.2)	117 (73.6)	175 (79.5)
Missing	-	6 (2.4)	2 (1.3)	1 (0.5)
**Preschool aged patients**	146	140	93	128
**Use of ICS, n**				
**Yes**	112	106	72	96
No	34	34	21	32
**Tapered ICS, n (%)**				
Yes^†^	-	21 (19.8)	20 (27.8)	19 (19.8)
No	-	82 (77.4)	50 (69.4)	76 (79.2)
Missing	-	3 (2.8)	2 (2.8)	1 (1.0)
**School aged patients**	182	180	104	160
**Use of ICS, n**				
**Yes**	140	139	87	124
No	42	41	17	36
**Tapered ICS, n (%)**				
Yes^‡^	-	24 (17.3)	20 (23.0)	25 (20.2)
No	-	112 (80.6)	67 (77.0)	99 (79.8)
Missing	-	3 (2.2)	0 (0.0)	0 (0.0)

*There were 45 patients who newly tapered their ICS at 4 weeks, 17 at 8 weeks and 9 at 12 weeks.

^†^There were 21 preschool aged patients who newly tapered their ICS at 4 weeks, 8 at 8 weeks and 3 at 12 weeks.

^‡^There were 24 school aged patients who newly tapered their ICS at 4 weeks, 9 at 8 weeks and 6 at 12 weeks.

### Treatment compliance and safety

Compliance with the treating regimen was high during the follow-up period with patients taking by average 91.6%, 93.6% and 92.2% of their prescribed doses upon 4, 8 and 12 weeks of treatment, respectively. During the course of the study, there were 182 non-serious adverse events (NSAEs) reported by 112 (34.1%) patients. Of these, 157 (86.3%) were probably or definitely not related to study medication and 15 led to study drug discontinuation in 12 (3.7%) patients. There were 25 (13.7%) NSAEs possibly, probably or definitely related to montelukast. Of these, the most frequent NSAEs related to montelukast were nightmares and sleep terror (n = 6), abdominal pain (n = 5), insomnia (n = 2) and headache (n = 2). A total of 3 serious adverse events (SAEs) were experienced by 3 patients: 1 asthma episode, 1 bronchitis and 1 pneumonia, none of which were judged to be related to the study medication by the treating physicians.

## Discussions

Although results from controlled randomized clinical trials indicate that montelukast is efficacious in the treatment of asthmatic children [13,14], continuous evaluation of the effectiveness and safety of montelukast in a less controlled real-life setting is essential in order to help health care professionals bridge the gap between current knowledge and practice in the management of asthmatic children. Accordingly, the principal objective of this study was to assess the effectiveness of montelukast administered either as a monotherapy or in combination with ICS treatment in children with uncontrolled asthma. Furthermore, in line with the fact that recommendations for asthma treatment differ according to children age categories [5,8,20], the effectiveness assessments of montelukast asthma treatment strategies were stratified by preschool and school aged pediatric patients.

The results of this 12-week multicenter observational study support the therapeutic effectiveness of montelukast in pediatric patients with uncontrolled asthma, in a clinical setting emulating real-life. Asthma control was achieved by the majority of patients who received montelukast either as monotherapy or in combination with ICS treatment for 12 weeks. Furthermore, clinically and statistically significant decreases in ACQ scores were observed after 4 and 12 weeks of treatment with montelukast mono- and add-on therapies, among both preschool and school-aged patients.

Although cross-study comparisons are difficult due to differences in study designs and diversity in efficacy outcomes, the results of the current study are consistent with the efficacy profiles of montelukast in childhood asthma that were previously reported in systematic reviews and randomized clinical trials conducted in preschool [11,13,21,22] and school-aged [11,14,22-28] children. Furthermore, the observed ACQ improvement is significantly higher to that observed with placebo in clinical trials with comparable follow-up schedules to the current study [29,30]. In addition, our findings provide further evidence of the benefits of montelukast administered either as monotherapy or in combination with ICS in everyday childhood asthma management and real-life clinical practices.

Asthma is the most common chronic disease of childhood and a leading cause of childhood morbidity. In addition to considerably affecting children’s physical, emotional and social lives, uncontrolled asthma also directly correlates with a loss of productivity and quality of life of the children’s caregivers [31,32]. Therefore, an effective strategy for the management of pediatric asthma should involve the development of an effective, convenient, safe and well tolerated pharmacologic intervention while improving the quality of life of the children and their caregivers.

The results of this study indicate that both asthmatic children and their caregivers can benefit from montelukast therapy since it is an effective treating option enabling asthma control, while significantly improving the caregivers’ quality of life. After 12 weeks of treatment with montelukast administered as monotherapy or in combination with ICS, clinically (mean change of ≥ 0.7 in PACQLQ score) and statistically (p < 0.001) significant improvements in caregivers’ quality of life were observed with mean (SD) changes in PACQLQ score of 1.25 (1.30) and 1.63 (1.32) from baseline, respectively.

Furthermore, the vast majority of the patients and physicians were satisfied or very satisfied with montelukast. This high level of satisfaction can be probably attributed to the observed effectiveness of montelukast in controlling asthma symptoms and improving caregivers’ quality of life, the ease of medication administration which enhances treatment compliance, and the safety and tolerability profile of montelukast.

The results of previous randomized clinical trials conducted with adult asthmatic patients suggested that montelukast could facilitate a reduction in ICS use [33,34]. However, the evidence on the ICS-sparing effect of montelukast in children is sparse and inconsistent. While Strunk RC *et al.*[35] reported that montelukast is not an effective ICS-sparing alternative in children, Tamesis GP *et al.*[36] have shown significantly lower use of supplemental ICS by children when montelukast was added to their ICS treatment. Moreover, although Phipatanakul W *et al.*[27] reported a non-significant reduction of ICS dose, they observed that children aged between 6 and 14 years experienced by average a 17% decrease in their ICS dose. In the current study the potential ICS-sparing effect of montelukast in children with uncontrolled asthma was also examined. In order to better reflect the everyday clinical practice, ICS tapering guidelines were distributed to treating physicians and the decision of tapering the ICS dosage was left to the discretion of the physician and made on an individual basis. After 12 weeks of treatment with montelukast in combination with ICS, 71 (21.6%) children reduced their ICS daily dosage. These results further reinforce the potential ICS-sparing benefit of montelukast in asthma childhood.

Overall, once-daily administration of montelukast for 12 weeks was well tolerated in the context of this study. The observed safety and tolerability results are consistent with the safety profile of montelukast previously reported in asthma childhood [2,13,14,22,24-26,37-39].

Potential limitations of the current study are related to the open-label and single cohort design without a parallel control group. However, since this study emulated the real-world clinical setting, blinding to the treatment used and comparison to a control group were not appropriate. Furthermore, the primary objective of the study was to assess the real-life effectiveness of treatment with montelukast in achieving asthma control and not the comparison of montelukast treatment with alternate treatment strategies. By conducting within- instead of between-group comparison, possible confounding bias related to disease and lifestyle factors that could affect the effectiveness of montelukast were minimized since each patient provided both control (pre-treatment) and on-treatment data. In light of the heterogeneous response documented for both ICS [39,40] and leukotriene receptor antagonist [39] treatments, and the real-life practice in which physicians often switch treatment in patients who are not responding or adhering to their current therapy, there may be concerns that the selected patients may have been more likely to respond to montelukast treatment. However, treatment response could not have been foreseen as no patient characteristics are currently known to predict response to montelukast [41]. The follow-up schedule recommended in the current study may not be representative of Canadian routine clinical practice which may have resulted in increased adherence with treatment and, thus, treatment effectiveness compared to that observed in real-life. Observational studies are required in order to substantiate this hypothesis. However, frequent assessment of uncontrolled asthma should be encouraged given that uncontrolled asthma has been shown to predict future risk of instability and exacerbations [42]. Finally, in the current analysis 92 (21.9%) patients who had an ACQ score of ≤ 0.75 at baseline were excluded. This was due to the fact that, although the primary outcome measure was the proportion of patients achieving asthma control based on the ACQ criteria, in order to be eligible for the study patients had to have uncontrolled asthma as per the Canadian Asthma Consensus Guidelines. However, it should be noted that both analyses gave comparable results.

An important strength of this study is the generalizability of its results to the Canadian target population. Since this study was conducted in a real-life clinical setting, inclusion and exclusion criteria were less selective and therefore more representative of the general population compared to the highly controlled environment of clinical trials. In addition, as recommended by the Global Initiative for Asthma (GINA), the current study focused on asthma control achievement rather than on asthma severity [2,5,8]. The effectiveness of montelukast in controlling asthma symptoms was assessed with the asthma control questionnaire (ACQ), a cost-effective [43] and validated questionnaire [15,16,43]. Since there are no reliable or validated measures of pulmonary airways function in preschool children younger than 6 years old [44] and given that the omission of the question on the forced expiratory volume from the seven-item ACQ does not alter the validity and the measurement properties of the instrument [45], the use of ACQ for assessing asthma effectiveness outcomes is considered suitable for the real-life clinical management of childhood asthma. Finally, the use of standardized and validated questionnaires to assess asthma control (ACQ) [15,16,43] and caregiver’s quality of life (PACQLQ) [17], also enhances the internal validity of the study.

## Conclusions

In conclusion, the results of this study indicate that montelukast, administered either as monotherapy or in combination with ICS treatment, is an effective, convenient and well tolerated therapeutic option for the management of asthma in preschool and school aged paediatric patients with uncontrolled asthma symptoms.

## Abbreviations

ICS: Inhaled corticosteroids; LTRAs: Leukotriene receptor antagonists; PEF: Peak expiratory force; SABA: Short-acting β2-agonist; LABA: Long-acting β2-agonist; ACQ: Asthma Control Questionnaire; PACQLQ: Pediatric asthma caregivers quality of life questionnaire; GINA: Global initiative for asthma; SD: Standard deviation; NSAEs: Non-serious adverse events; SAEs: Serious adverse events.

## Competing interests

DB has received honorariums for consulting (Ad Board) and lecturing (CME) from Abbott, Altana, AstraZenaca, Graceway, GlaxoSmithKline, Merck, Novartis, Nycomed and Pfizer. MD is an employee of Merck Canada Inc. JSS is an employee of JSS Medical Research, the CRO contracted by Merck Canada Inc. to conduct the data management and analysis. AB has received research funding from CIHR, NSERC, AllerGen NCE and unrestricted educational grants from AZ, Graceway, GSK, Merck, Novartis and Nycomed.

## Authors’ contributions

DB contributed to, interpretation, and the critical revision of the article. MD contributed to study design, interpretation, and the critical revision of the article. JSS contributed to study design, data analysis, interpretation, drafting the manuscript, and the critical revision of the article. AB contributed to data collection, interpretation, and the critical revision of the article. All authors have approved the current version of the manuscript.
